# Increasing the adoption of electric vehicles may exacerbate carbon emissions from power plants in China

**DOI:** 10.1016/j.isci.2026.116710

**Published:** 2026-07-27

**Authors:** Wenqian Ren, Jing Liang, Xu Peng

**Affiliations:** 1 School of Economics and Management, Harbin Institute of Technology, Harbin, Heilongjiang, China; 2 School of Business, Jiangnan University, Wuxi, Jiangsu, China

**Keywords:** electric vehicles, power plants, carbon emissions, clean energy

## Abstract

China has committed to electric vehicle (EV) development to achieve the “Dual Carbon” goals. However, the rising electricity demand from EVs may exacerbate carbon emissions from the power sector. This empirical study investigates the impact of China’s EV adoption on carbon emissions in the power sector using the panel data from 2017 to 2022. The instrumental variable regression results reveal that a 10% increase in EV sales increases emissions from power plants by 0.39%. Spatial econometric results further indicate significant emission spillovers and spatial connections among power plants. In addition, our scenario analysis reveals that the coordinated development of renewable energy and transport electrification contributes further to carbon reduction. Our findings highlight the importance of a cleaner energy mix in unlocking the full decarbonization potential of EVs.

## Highlights

• EV adoption links transport electrification with power-sector carbon emissions
• A 10% rise in EV sales raises power plant emissions by 0.39%
• Electricity transmission generates spatial spillovers in carbon emissions
• The development of clean energy unlocks the decarbonization potential of EVs

## Graphical abstract

**Figure:**
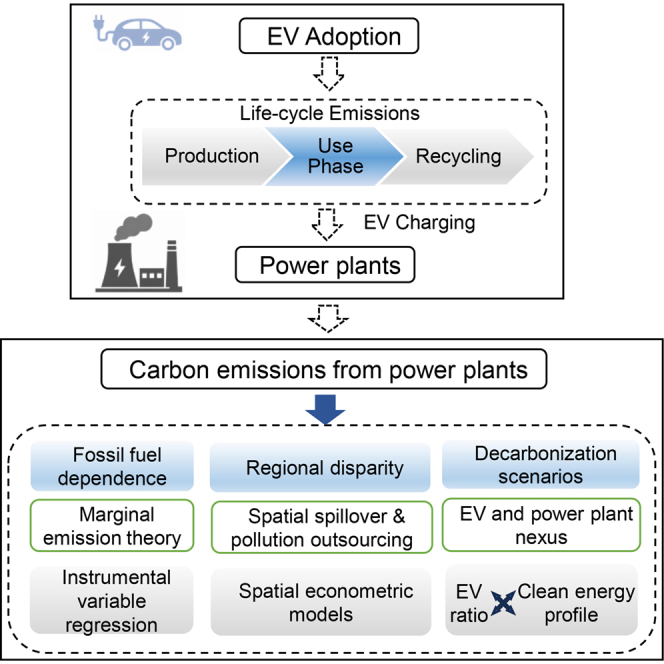

## Teaser

Applied sciences; Electrical engineering; Power engineering

## Introduction

The global shift toward electric mobility has become a pivotal strategy in addressing climate change and environmental pollution.^1^ Rapid urbanization, rising energy demands, and increasing carbon emissions underscore the need for sustainable transportation solutions.^2^ Replacing internal combustion engine vehicles (ICEVs) with electric vehicles (EVs) in China, the world’s largest automobile market, is vital for its goal of carbon neutrality.^3^^,^^4^ By eliminating tailpipe emissions, EVs demonstrate substantial potential for mitigating environmental pollution and carbon emissions within the transport sector.^5^^,^^6^ However, EVs’ dependence on electricity generation from fossil power plants can result in considerable indirect emissions, which may undermine their environmental advantages.^7^ Nevertheless, China’s current energy infrastructure remains predominantly coal-dependent, with thermal power responsible for over 60% of national electricity generation.^8^ The exponential growth in EV adoption, directly leading to heightened electricity demand, may amplify emissions from carbon-intensive power generation in China.^9^^,^^10^

Empirical research has demonstrated that the transition from ICEVs to EVs can yield significant emissions abatement.^11^ The life cycle assessment indicates that EVs can decrease greenhouse gas emissions by 30–60% relative to gasoline-powered vehicles.^12^ EVs generally consume less energy during operation, particularly when charged with clean energy. If fossil-fuel power generation serves as the primary source to meet the electricity demand, the environmental benefits of EVs will be greatly compromised.^1^^,^^13^^,^^14^ Existing studies have examined the role of energy mix in shaping the overall emissions from EVs and have found that while EVs reduce carbon emissions in countries with cleaner power grids, their advantages are limited in regions with a greater reliance on fossil fuels.^8^^,^^15^ This underscores the importance of transitioning to cleaner energy sources.

Currently, the rising adoption of EVs imposes rapidly increasing demands on the power infrastructure.^16^ As the number of EVs grows, the electricity demand increases, which may pose a higher load on the power grid.^17^ Recent studies further highlight the importance of accurately forecasting EV charging demand for effective grid management, emphasizing the complex spatiotemporal patterns of charging behavior and their implications for system operations.^18^ In China, managing the electricity flow from areas with abundant renewable energy resources to regions reliant on coal-based power generation is critical for optimizing the emissions benefits of EV adoption.^19^ Therefore, an empirical analysis considering the transport and power sectors is essential for accurately assessing EVs’ decarbonization potential.

**Figure 1:**
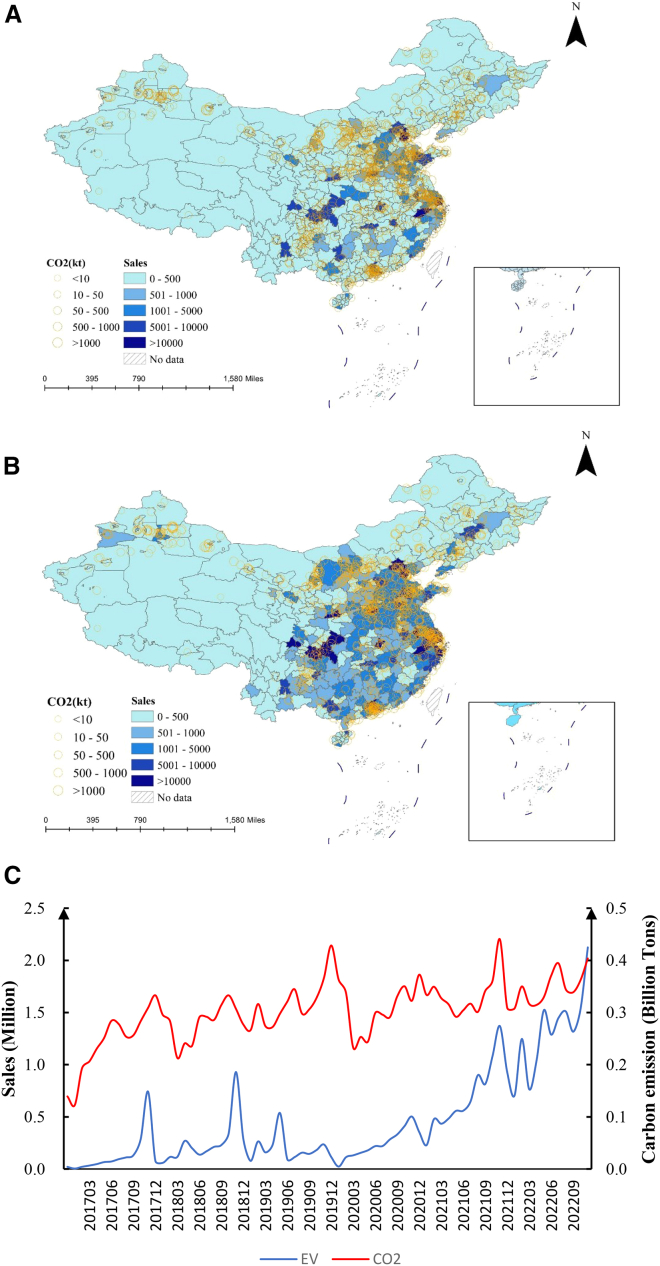
Spatial distribution and temporal trends of EV sales and carbon emissions in China (A) EV sales and power plant carbon emissions in 2017. (B) EV sales and power plant carbon emissions in 2022. (C) Monthly trends of EV sales and carbon emissions.

There is a positive regional correlation between EV sales and carbon emissions from the energy sector (Figures 1A and 1B). The eastern regions generally exhibit higher EV sales and carbon emissions than the western regions. From 2017 to 2022, China witnessed substantial growth in urban EV sales (c). The sharp rise in 2021 reflects the rapid growth, likely driven by governmental policies and market demand.^20^ Carbon emissions exhibit a general upward trend, with noticeable peaks corresponding to spikes in EV sales.

Against the backdrop of China’s “Dual Carbon” goals, understanding the net environmental consequences of transport electrification requires moving beyond the transport sector and explicitly accounting for the power system that supports EV deployment. While existing studies largely emphasize tailpipe emission reductions, they often overlook the indirect carbon impacts transmitted through electricity generation, as well as the spatial interdependence inherent in power systems. This study fills these gaps by providing a causal and spatially explicit the assessment of the impact of EV adoption on power-sector carbon emissions in China. Methodologically, we integrate instrumental variable (IV) and spatial econometric approaches to address endogeneity arising from spatial correlation and unobserved regional heterogeneity, and further validate the results using double machine learning (DML) techniques. Finally, through scenario analysis, we demonstrate that the carbon outcomes of EV expansion critically depend on the cleanliness of the electricity mix, underscoring that the climate benefits of transport electrification can only be realized in tandem with sustained decarbonization of the power sector.

This paper investigates the relationship between EV adoption and carbon emissions from power plants. The study employs a two-way fixed-effects model with IVs to address endogeneity and applies spatial econometric methods to identify spillover effects in power grid operations. The empirical model is extended through a scenario-based assessment, projecting the combined impacts of future EV adoption and grid decarbonization on carbon emissions. We find that EV adoption increases carbon emissions in the power sector. The findings highlight the crucial importance of a cleaner energy mix in unlocking the full decarbonization potential of transport electrification.

## Results

### EVs’ impact on carbon emissions from thermal power plants

The regression results in Table 1 show that EV adoption increases carbon emissions from power plants. Columns (1) and (2) are benchmark regressions without addressing endogeneity, while columns (3) and (4) apply the IV approach. These models include fixed effects for power stations, months, and years to control for unobserved heterogeneity at these levels. In columns (1) and (2), the coefficients for EV sales are positive and statistically insignificant. Columns (3) and (4) indicate that a 10% increase in EV adoption increases carbon emissions from power plants by 0.39%. These results suggest that, after addressing potential endogeneity, an increase in EV sales intensifies carbon emissions from thermal power plants. The IV is the product of the logarithms of EV charging station numbers and the road lengths of cities. Improving EV infrastructure promotes EV adoption by mitigating range anxiety and reducing perceived adoption barriers,^13^^,^^21^ but does not directly affect power plants’ carbon emissions. This IV is valid and passes the under identification test and weak identification test. In column (4), the results underscore a critical paradox: while EVs reduce direct on-road emissions, they may increase fossil fuel consumption in the power sector under the current energy structure. The carbon reduction targets necessitate coordinated strategies between transportation and power sectors.^22^

**Table 1:** Benchmark regression and IV regression

Variables	(1)	(2)	(3)	(4)
	lnCO_2_	lnCO_2_	First-stage	Second-stage
EV sales	0.00177 (0.00314)	0.000928 (0.00319)		0.0391∗∗ (0.0196)
Charging × road			0.190∗∗∗	
			(0.0196)	
Controls	N	Y	Y	Y
Power plants FE	Y	Y	Y	Y
Month-by-year FE	Y	Y	Y	Y
K-P rk LM	–	–	18.291	
K-P rk Wald F	–	–	94.920	
Observations	55,369	55,369	55,369	55,369
Power plants	845	845	845	845

Robust standard errors in parentheses; Clustered at the power plant level. ∗∗∗*p* < 0.01, ∗∗*p* < 0.05, and ∗*p* < 0.1.

Besides, we conduct a series of sensitivity analyses reported in Table S7. Specifically, we apply alternative winsorization thresholds and augment the fixed effects specification by including province-by-year fixed effects. The estimated coefficients remain positive and statistically significant across these specifications, suggesting that the main results are robust to alternative treatments of outliers and model specifications.

We employ DML approaches to support the IV regression results. The estimated coefficients using random forest, support vector machine, gradient boosting, and neural network are positive and statistically significant, reinforcing the finding that EV adoption exacerbates carbon emissions from power plants (Table S4). ML methods are particularly well-suited for capturing complex nonlinear relationships and high-dimensional interactions among variables.^.^^23^ These models uncover complex relationships in the data without imposing rigid functional form assumptions. The consistency of results across different algorithms enhances the robustness and credibility of our findings.

We also test the impact of EV sales on nitrous oxide (N_2_O), methane (CH_4_), and carbon emissions in the database of Multi-resolution Emission Inventory for China (MEIC) (Table S5). These results confirm that the relationship between EV adoption and emissions applies to alternative emissions and provide evidence that the results are not driven by a particular data source, further strengthening the validity of the findings.

### Mechanism analysis

Table 2 reveals that increased EV penetration leads to higher power sector emissions by amplifying fossil-fuel electricity production. This two-stage mediation analysis identifies fossil-fuel electricity generation as the mediating mechanism. Column 1 confirms the direct positive association between EV adoption and carbon emissions, while column 2 demonstrates that EV adoption significantly increases fossil-fuel electricity generation. This aligns with related literature that suggests an increasing supply of fossil-fuel electricity exacerbates carbon emissions in the power sector.^24^

**Table 2:** Mechanism analysis

Variables	(1)	(2)	(3)
	Main regression	Mechanism	Mechanism
	lnCO_2_	Fossil_electricity	Power_import
EV sales	0.0391∗∗ (0.0196)	0.0679∗∗∗ (0.0160)	0.557∗∗∗ (0.167)
Controls	Y	Y	Y
Power plants FE	Y	Y	Y
Month-by-year FE	Y	Y	Y
IV	Y	Y	Y
Observations	55,369	55,369	55,369
Power plants	845	845	845

Robust standard errors in parentheses; clustered at the power plants level. ∗∗∗*p* < 0.01, ∗∗*p* < 0.05, and ∗*p* < 0.1.

To further deepen the mechanism analysis, we examine whether EV adoption also affects electricity system operations through changes in interregional power flows. As shown in column (3), EV sales significantly increase power imports, indicating that the additional electricity demand induced by EV charging is not fully satisfied by local generation but is partly met through interprovincial electricity transmission. This finding highlights an additional channel linking EV diffusion to carbon emissions, whereby demand-side electrification leads to the spatial reallocation of electricity generation and associated emissions across regions.

The increases in both fossil-fuel electricity generation and power imports suggest that the emissions effects of EV adoption are closely linked to adjustments in the marginal electricity supply. Rising EV-related electricity demand, particularly under heterogeneous and potentially concentrated charging patterns, may increase peak-load pressure on local power systems and require additional electricity imports from other regions.^25^^,^^26^ These patterns are consistent with a demand-driven dispatch response in which incremental electricity demand is met by carbon-intensive generation sources within the local grid or through cross-regional electricity transmission.^27^ Although high-frequency charging behavior and dispatch decisions cannot be directly observed in our data, the evidence suggests that electricity system adjustments play an important role in shaping the observed emissions impacts. Overall, the findings indicate that EV adoption affects carbon emissions from power plants through both increased fossil-fuel-based electricity generation and adjustments in interregional electricity allocation.

### Spatial dependence

Power stations are usually connected, and geographically proximate power stations may exhibit spatial correlation. Carbon emissions from one plant are influenced by neighboring plants through shared grid infrastructure, inter-regional electricity trading, and technology diffusion.^28^ Technology diffusion includes the spillover of both efficiency-enhancing generation technologies (e.g., ultra-supercritical units) and carbon abatement technologies (e.g., flue gas desulfurization and carbon capture), propagating through learning, benchmarking, and policy coordination. To comprehensively assess the net environmental impact of EVs, it is essential to account for these spatial dependencies. This paper employs spatial regression models to address spatial dependence.^29^

Table 3 presents the spatial regression analysis results, which account for spatial dependencies between power stations. We report results for the spatial autoregressive model (SAR) and the spatial error model (SEM), using two spatial weight matrices: inverse distance and binary contiguity. The SEM model is preferred because the *p* value of the LM test is less than 1% (Table S6). In columns (1) and (2), the spatial weight matrix is based on the inverse distance between power stations. In columns (3) and (4), the spatial weight matrix is based on the power grid proximity, where a value of 1 is assigned if two power plants are spatially adjacent.

**Table 3:** Results using spatial regressions

Variables	(1)	(2)	(3)	(4)
	Inverse distance		Binary contiguity	
	SAR	SEM	SAR	SEM
EV sales	0.00505∗∗ (0.00218)	0.00915∗∗ (0.00460)	0.00272 (0.00226)	0.00805∗ (0.00412)
rho	0.792∗∗∗ (0.0324)		0.816∗∗∗ (0.0157)	
lambda		0.799∗∗∗ (0.0318)		0.828∗∗∗ (0.0155)
Controls	Y		Y	
Power plant FE	Y		Y	
Month-by-year FE	Y		Y	
Observations	18,216	18,216	18,216	18,216
Power plants	253	253	253	253

Robust standard errors are in parentheses; Clustered at the power plants level. ∗∗∗*p* < 0.01, ∗∗*p* < 0.05, and ∗*p* < 0.1.

The estimated SAR coefficient (rho) is positive and highly significant across specifications, indicating strong spatial spillovers in power plant carbon emissions. This suggests that emission changes at one power plant are systematically transmitted to neighboring plants through the interconnected power system. Moreover, the spatial error coefficient (lambda) is also positive and statistically significant. This finding implies the presence of spatially correlated unobserved factors, such as regional electricity dispatch policies, grid characteristics, and local energy structures, which constitute an important source of endogeneity in the benchmark regression.

The coefficients on EV sales are positive in all models, indicating that after accounting for spatial dependencies, an increase in EV sales leads to higher carbon emissions from power stations. Our results indicate significant spatial dependence in the emissions of power plants, and that EV adoption increases carbon emissions from plants by 0.92%. The spatial econometric analysis supports the IV regression findings while providing a means to address spatial endogeneity. The spatial econometric analysis also confirms that spatial dependence may contribute to endogeneity in the benchmark regression model. Our results highlight that recognizing the influence of interregional electricity exchanges on emissions is crucial for addressing endogeneity and ensuring the robustness of the empirical analysis.

### Regional disparity

**Figure 2:**
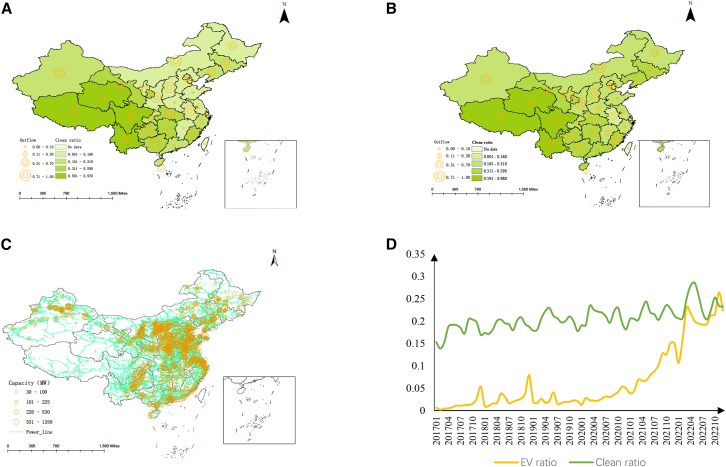
The overview of clean energy development and regional electricity transmission (A) The electricity outflow ratio and clean grid ratio at China’s provincial level, 2017. (B) The electricity outflow ratio and clean grid ratio at China’s provincial level, 2022. (C) The power grid and power plants. (D) The time trend of the EV ratio and clean electricity ratio.

There are regional disparities in electricity transmission and clean energy generation in China (Figure 2). Regions endowed with abundant renewable resources, such as hydropower and solar energy, often produce surplus electricity, typically resulting in net outflows. Specifically, provinces such as Tibet and Yunnan, which are rich in clean energy, exhibit higher electricity outflow ratios. In contrast, provinces such as Guangdong and Beijing have much lower outflow ratios. This regional disparity highlights a critical challenge: Clean energy production is geographically concentrated, making its transmission to high-demand areas essential for the decarbonization of the power sector.^30^^,^^31^ C shows the locations and capacities of thermal power plants and depicts the interconnection structure via the power grid. We find that EV adoption mainly exacerbates the carbon emissions from the low-capacity (<300 MW) power plants because their decarbonization standards are lower than those of the high-capacity plants (Table S3).

The regression results (Table 4) indicate significant regional heterogeneity in the impact of EV sales on carbon emissions from power plants. In the western region, EV adoption yields the greatest environmental benefit, reducing emissions by 4.49%. However, in the central region, EV sales are positively and significantly correlated with emissions, increasing them by 33.3%. This suggests that increased electricity demand from EVs in these areas may still be met primarily by fossil fuel-based generation, such as coal. Our findings suggest that more developed cities may outsource environmental costs to coal-dependent inland regions via interprovincial electricity transmission, which may raise concerns about environmental justice and spatial equity. This pattern highlights potential concerns regarding spatial justice, as regions with cleaner energy structures benefit from EV adoption while coal-dependent regions bear a disproportionate share of the associated emissions. It also points to the need for inter-regional eco-compensation mechanisms to address the uneven distribution of environmental costs arising from electricity transmission.

**Table 4:** Regional heterogenicity

Variables	(1)	(2)	(3)
	Eastern region	Central region	Western region
EV sales	0.00284 (0.00307)	0.333∗∗∗ (0.0895)	−0.0449∗∗ (0.0215)
Controls	Y	Y	Y
Power plant FE	Y	Y	Y
Month-by-year FE	Y	Y	Y
IV	Y	Y	Y
Observations	25,652	12,678	10,657

Robust standard errors are in parentheses; clustered at the power plant level.∗∗∗*p* < 0.01, ∗∗*p* < 0.05, and ∗*p* < 0.1.

We also examine the heterogeneity among regions with different clean energy ratios. The results reveal that EVs lead to more carbon emissions in regions with a lower clean energy ratio (Table S2). These findings underscore the importance of regional energy structures in shaping the environmental outcomes of transport electrification. Moreover, EV adoption contributes to higher carbon emissions in high-income provinces (Table S3), where residents possess greater purchasing power and are more likely to adopt EVs. The rising EV adoption increases the electricity demand for charging. In provinces with high electricity demand, reliance on fossil fuels may persist due to limited access to imported clean energy. Strengthening interprovincial electricity exchange, improving transmission networks, and enhancing cross-regional coordination can reduce dependence on carbon-intensive generation and support a cleaner national energy system.^32^^,^^33^

### Decarbonization scenarios

The scenario analysis examines the interplay between EV adoption and clean energy integration under varying conditions, providing insights into the potential carbon emission outcomes and policy implications. EV penetration and the clean energy ratio in China increased significantly from 2017 to 2022 (Figure 2D). These historical trends provide the foundation for the scenario analysis of future carbon emissions. We build future scenarios based on the Announced Pledges Scenario (APS) from the IEA (2021) and China’s New Energy Vehicle Industry Development Plan (2021–2035).^34^ The EV penetration rates for 2035 and 2050 are directly taken from projections implied by China’s New Energy Vehicle Industry Development Plan, while the clean energy ratios are adopted from the International Energy Agency under China’s APS. These scenario values are based on external policy and scenario projections and are used here for illustrative analysis rather than independent forecasting.

**Figure 3:**
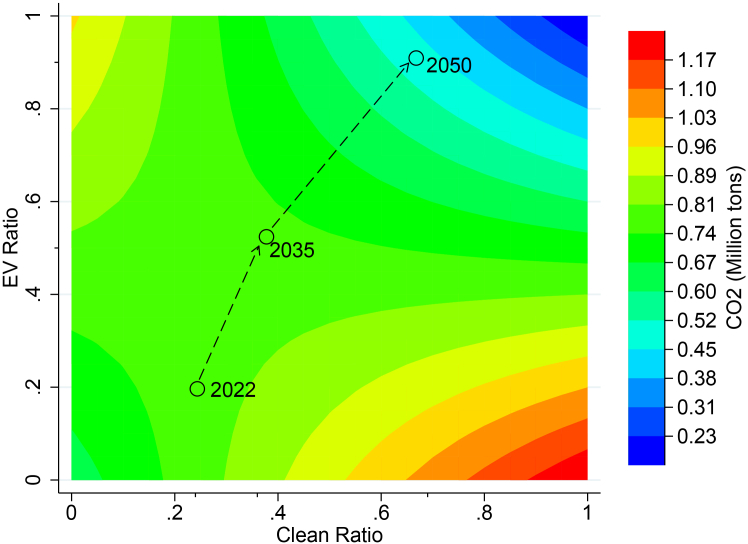
The decarbonization path of power plants

There are three scenarios for grid decarbonization: low (clean ratio<20%), moderate (20–50% clean ratio), and high (clean ratio>50%), and three scenarios for EV development: low (<20% EV sales), moderate (20%–50% EV sales), and high (>50% EV sales). We would expect that in a low-decarbonization scenario, high EV adoption increases emissions due to fossil fuel reliance.^35^^,^^36^ In contrast, a highly decarbonized grid enables net carbon reductions, making EV adoption an effective decarbonization strategy. The results (Figure 3) show that high EV adoption increases emissions in a low-decarbonization scenario and EV adoption reduces emissions effectively in a high-decarbonization scenario, particularly in renewable-rich regions.

From 2022 to 2050, as the EV ratio increases from 0.20 to 0.90 and the clean energy ratio rises from 0.24 to 0.74, total carbon emissions decline by approximately 53%, from 0.85 to 0.40 billion tons. Note that the most significant reduction in emissions occurs when the clean energy ratio exceeds 0.6, corresponding to the transition into the blue-shaded region. In 2035, with a clean grid ratio of 36% and an EV proportion of 50%, the power sector could achieve substantial emission reductions. In 2050, with a clean ratio of 69% and an EV proportion of 90%, net-zero emissions are likely to be achieved. However, the APS scenario is not the optimal decarbonization path. If China can accelerate its energy transition and align the clean energy ratio with the growth of EVs, it will achieve its carbon reduction goals more rapidly.

## Discussion

This study examines the impact of EV adoption on carbon emissions in China’s power sector. Our regression results show that the rising number of EVs, through increased electricity demand, leads to higher emissions from power generation. The spatial econometrics analysis indicates that neighboring power plants exchange electricity and lead to carbon emission transfers through the power grid. Our findings highlight the critical need to decouple EV growth from reliance on high-carbon energy sources. A cleaner grid would allow for the potential of EVs to be harnessed in mitigating the effects of climate change.

Moreover, this paper shows that EV adoption increases fossil-fuel-related electricity consumption. This implies that coal-dependent areas should prioritize renewable and clean energy development. These findings also highlight the importance of addressing spatial equity concerns, as these regions may bear a disproportionate share of the environmental burden associated with electricity generation. In this context, inter-regional eco-compensation mechanisms and coordinated policy frameworks could help to balance the distribution of costs and benefits across regions. Furthermore, energy storage development and smart grid technologies are essential to manage the increased electricity demand.^37^ These technologies would facilitate the integration of renewable energy into the grid, ensuring that the growth in EV numbers does not outpace the reduction in grid emissions.^38^ The coordinated investment in clean energy and energy infrastructure is crucial for strengthening the decarbonization effects of EV adoption.

The policy implications are as follows. First, measures should be taken to encourage EV adoption combined with clean energy. Investment in solar, wind, and hydropower improves the clean energy ratio and contributes to achieving the carbon neutrality goal.^39^ The development of clean energy promotes the carbon reduction benefits of EVs. To further enhance this effect, governments should implement policies that incentivize the expansion of renewable energy infrastructure. Second, we should improve energy storage technologies, and the battery circular economy can enhance the efficiency and reliability of renewable energy utilization. Third, policy design should promote power transmission between regions. Improving interprovincial transmission capacity is crucial for harnessing clean energy nationwide.^40^ In addition, deploying energy storage systems and demand response programs can better align EV charging with clean electricity generation. The power transmission and storage infrastructure provide carbon reduction potential for EV adoption.^41^ Moreover, the development of electricity spot markets and green electricity trading can provide real-time price signals that reflect the marginal carbon intensity of power generation, thereby incentivizing EV charging to shift toward low-emission periods. Such market-based mechanisms can improve the temporal alignment between electricity demand and renewable supply, enhancing the overall decarbonization impact of EV adoption. While these implications are suggestive, they should be interpreted with caution, as they extend beyond the direct empirical evidence presented in this study.

### Limitations of the study

This study has several limitations that should be acknowledged. First, carbon emission data in this paper were derived from the power-sector dataset of the Emissions Database for Global Atmospheric Research (EDGAR) and matched to power plants by geographic location, which may introduce measurement errors. Although we mitigate potential bias through multiple empirical strategies and robustness checks, future studies could benefit from more precise plant-level carbon emission data.

Second, given the limited availability of city-level peak electricity demand data, we only indirectly assess the impact of EV adoption on peak electricity demand through interprovincial electricity transmission. Future research could incorporate higher-frequency electricity load data to identify how EV charging demand affects peak electricity demand and power-sector carbon emissions.

Third, the scenario analysis in this paper relies on projected EV sales and energy mix derived from existing studies rather than from an integrated forecasting framework. Future research could develop a comprehensive modeling framework that jointly projects EV adoption, electricity demand, renewable energy penetration, and carbon emissions. The theoretical and empirical findings of this study may provide a useful foundation for such integrated assessments.

## Resource availability

### Lead contact

Further information and requests for resources and reagents should be directed to and will be fulfilled by the lead contact, Jing Liang (j.liang@hit.edu.cn).

### Materials availability

This study did not generate new unique reagents.

### Data and code availability

• Carbon emission data were obtained from the Emissions Database for Global Atmospheric Research (EDGAR) at https://edgar.jrc.ec.europa.eu/. Data on EV adoption were collected from compulsory traffic insurance statistics reported by the China Association of Automobile Manufacturers at http://www.caam.org.cn/. The socio-demographic data, including GDP, population, and road length, were obtained from the National Bureau of Statistics of China at https://www.stats.gov.cn/english/. Meteorological data, including average temperature, standard pressure, wind speed, and precipitation, were retrieved from the National Centers for Environmental Information (NCEI) at https://www.ncei.noaa.gov/. Data processing and statistical analyses were conducted using Stata and R software. Spatial visualization and map generation were performed using ArcGIS 10.8.
• All custom code is available on Zenodo at https://doi.org/10.5281/zenodo.20744413.
• Any additional information required to reanalyze the data reported in this paper is available from the lead contact upon request.

## Acknowledgments

This work is supported by funding from the 10.13039/501100001809National Natural Science Foundation of China (grant nos. 72574051 and 72304112).

## Author contributions

W.R.: project development, data collection, data analysis, manuscript writing. J.L.: project conception and development, data analysis, research design, manuscript editing, funding acquisition. X.P.: data collection, data analysis, manuscript editing, funding acquisition.

## Declaration of interests

The authors declare no competing interests.

## STAR★Methods

### Key resources table

**Table:** 

REAGENT or RESOURCE	SOURCE	IDENTIFIER
**Deposited data**		

Carbon emission	Emissions Database for Global Atmospheric Research	https://edgar.jrc.ec.europa.eu/
EV number	Compulsory traffic insurance data from the Public Security Database	http://www.caam.org.cn/
GDP	National Bureau of Statistics	https://www.stats.gov.cn/english/
Population	National Bureau of Statistics	https://www.stats.gov.cn/english/
Average temperature	National Centers for Environmental Information	https://www.ncei.noaa.gov/
Standard pressure	National Centers for Environmental Information	https://www.ncei.noaa.gov/
Wind speed	National Centers for Environmental Information	https://www.ncei.noaa.gov/
Precipitation	National Centers for Environmental Information	https://www.ncei.noaa.gov/
Electricity flow-in	National Bureau of Statistics	https://www.stats.gov.cn/english/
Electricity flow-out	National Bureau of Statistics	https://www.stats.gov.cn/english/
Charging station	Baidu Map	https://map.baidu.com/
Road length in 2001	National Bureau of Statistics	https://www.stats.gov.cn/english/
Fossil fuel electricity	National Bureau of Statistics	https://www.stats.gov.cn/english/
Code for empirical analysis	This paper	https://doi.org/10.5281/zenodo.20744413

**Software and algorithms**		

ArcGIS 10.8	ESRI	https://www.arcgis.com/index.html
Stata software	Stata	https://www.stata.com/
R version 4.4.2	RStudio, PBC	https://www.rstudio.com/

### Method details

#### Data sources and key variables

The sales of EVs during 2017–2022 at the city level are obtained from the compulsory traffic insurance database from the Chinese Public Security, which closely approximates actual EV sales. The thermal power plant data is sourced from the Global Coal Plant Tracker, which includes the capacity, location (latitude and longitude), and operating status of all the power plants in China. The data source of carbon emissions is the 0.1 ° × 0.1 ° grid emission map in the Emissions Database for Global Atmospheric Research (EDGAR),^42^ including carbon dioxide, methane, and nitrogen oxides. This paper matches the location of each power station with the closest grid point from the EDGAR power sector emissions database. As the EDGAR dataset provides sector-specific, high-resolution estimates of power-sector emissions, this proximity-based matching offers a reasonable approximation of plant-level emission intensity, although some measurement error may remain. Then, this paper matches the power plants with the EV sales in each city. The source of socioeconomic data is the National Bureau of Statistics (NBS). The meteorological control variables are from the National Centers for Environmental Information (NCEI) (Table S1).

#### Empirical strategies

##### Benchmark regression

This study examines the impact of EV adoption on carbon emissions from power stations in China using a two-way fixed effects regression model. The model is as follows:

(Equation 1) $${lnEmissions}_{ict}=\beta {lnEV}_{ct}+\gamma Z_{ct}+\sigma_{i}+\mu_{t}+v_{it}$$

Equation 1, *i*, *c*, and *t* represent power stations, cities, and months from January 2017 to December 2022. *lnEV* is the logarithm of EV sales. *lnEmissions* denotes the logarithm of power plants’ carbon emissions. *Z* represents control variables, including GDP, population, meteorological factors, and electricity transmission flows. This model allows for a comprehensive analysis of how EV sales influence power sector emissions, accounting for temporal and spatial aspects.

##### Instrumental approach

This paper employs the IV approach to address the endogeneity in the benchmark model.

(Equation 2) $${lnEV}_{ct}=\beta {Instrument}_{ct}+\gamma Z_{ct}+\sigma_{i}+\mu_{t}+v_{it}$$

(Equation 3) $${lnEmissions}_{ict}=\beta \hat{{lnEV}_{ct}}+\gamma Z_{ct}+\sigma_{i}+\mu_{t}+v_{it}$$

In Equations 2 and 3, the instrument is the product of the logarithms of provincial EV charging station numbers and the cities’ road lengths in 2001. It is a proxy for infrastructure-related factors correlated with EV adoption but not directly with power station emissions. Equation 2 is the first-stage estimation of *lnEV* by the instrument. Equation 3 indicates the second-stage regression using the estimation from Equation 2. To further assess the validity of the instrument, Table S8 in the Supplementary Materials reports additional tests of the exclusion restriction. In particular, we estimate reduced-form specifications and examine whether the direct effect of the instrument on emissions diminishes once EV adoption is controlled. The results show that the coefficient on the instrument becomes smaller and less precisely estimated after controlling EV sales, suggesting that its effect on emissions operates through EV adoption. These findings provide supportive, though not definitive, evidence for the plausibility of the exclusion restriction. In addition, we construct an alternative instrument by interacting lagged EV sales with road length to further examine the robustness of our identification strategy. The results, reported in Table S9, remain qualitatively consistent with the baseline estimates, reinforcing the credibility of our IV approach.

##### Spatial econometrics

The spatial dependence of EV sales and carbon emissions may cause endogeneity in the benchmark regression. Therefore, this paper chooses the spatial models to identify the effects of EV adoption on carbon emissions and support the IV regression results.

$${lnEmissions}_{ict}=\beta {lnEV}_{ct}+\gamma Z_{ct}+\sigma_{i}+\mu_{t}+v_{it}$$

(Equation 4) $$v_{it}=\lambda {Wv}_{it}+u_{it}$$

(Equation 5) $${lnEmissions}_{ict}=\rho {WlnEV}_{ict}+\beta {lnEV}_{ct}+\gamma Z_{ct}+\sigma_{i}+\mu_{t}+v_{it}$$

Equation 4 indicates the spatial error model (SEM), and Equation 5 indicates the spatial autoregressive model (SAR). In these models, *W* is the spatial weight matrix. This paper employs two spatial weight matrices: inverse distance and binary contiguity. The inverse distance matrix is adopted to capture spatial dependence that attenuates with geographical distance, whereas the binary contiguity matrix reflects spatial interactions arising from geographical adjacency between power plants. The application of both matrices allows for a robustness examination of spatial spillover effects under alternative specifications of regional connectivity.

##### Mechanism

EV adoption increases electricity demand. This study examines the mediating effects in this pathway.

(Equation 6) $${lnFossilele}_{ct}=\beta {lnEV}_{ct}+\gamma Z_{ct}+\sigma_{i}+\mu_{t}+v_{it}$$

Equation 6, *lnFossilele* represents the fossil-fuel electricity. This equation examines the effects of EV adoption on fossil fuel consumption. Besides, our research employs the IV to identify the causal inference of EV adoption on fossil fuel. This process solves the potential endogeneity problems. Then, our study proves that fossil fuel consumption increases carbon emissions according to the related literature. This two-step approach explores the mediating effects of EV adoption on carbon emissions from power plants.

##### Scenario analysis

We employ a scenario analysis framework to evaluate the potential carbon emission outcomes under varying combinations of electric vehicle (EV) penetration and clean energy integration at the city level.

(Equation 7) $${lnEmissions}_{ct}=\beta {EV}_{ct}^{ratio}+\alpha {Clean}_{ct}^{ratio}+\omega {EV}_{ct}^{ratio}\times {Clean}_{ct}^{ratio}+\gamma Z_{ct}+\sigma_{c}+\mu_{t}+v_{c}t$$

Equation 7, ${EV}_{ct}^{ratio}$ represents the EV ratio in the market of one city, and ${Clean}_{ct}^{ratio}$ represents the clean energy ratio in electricity generation at the city level. *lnEmissions*_*ct*_ is the carbon emissions of power plants at the city level. The interaction term allows us to explore whether the emissions impact of EV adoption depends on the clean energy ratio. *σ*_*c*_ and *μ*_*t*_ represent the city fixed effects and time fixed effects.

### Quantification and statistical analysis

We use a two-way fixed-effects model to examine how EV adoption affects carbon emissions from power plants in China. We further apply instrumental variable (IV) regression to address potential endogeneity concerns. To identify spatial dependence and emission spillover effects among power plants, we estimate spatial autoregressive and spatial error models. We conduct heterogeneity analysis across regions and seasons and perform mechanism analysis to examine the roles of interprovincial electricity transmission, electricity market structures, and renewable energy development in shaping the relationship between EV adoption and power-sector carbon emissions. We conduct the statistical analysis using Stata 18. We use ArcGIS 10.8 for spatial visualization and map generation. Statistical details, including sample sizes, model specifications, and significance levels, are reported in the corresponding figures, tables, legends, and supplementary materials.
