# Supplementary material for: Increasing the adoption of electric vehicles may exacerbate carbon emissions from power plants in China
Source: iScience. 2026 Jul 27;29(8):116710. doi: 10.1016/j.isci.2026.116710 (PMC13429923; doi:10.1016/j.isci.2026.116710)
Supplement: Document S1. Tables S1–S9 [file mmc1.pdf]

iScience, Volume *29*

Supplemental information

Increasing the adoption of electric vehicles may exacerbate carbon emissions from power plants in China

Wenqian Ren, Jing Liang, and Xu Peng

1. Data and measurement

Table S1. Descriptive statistics

	Meaning	Data Source	Observations	Mean	Sd	Min	Max
CO_2	Carbon Emission (10^3 Tons)	EDGAR	55369	394.52	434.12	0.88	4989.36
sales	EV Number	Compulsory traffic insurance data from the Public Security Database	55369	595.40	1498.33	0.00	30120
gdp	GDP (10^8 Yuan)	NBS	55369	4389.47	4722.54	141.53	32387.68
popu	Population (10^4)	NBS	55369	496.21	290.83	21.00	2095.00
temp	Average Temperature (°F)	NCEI	55369	57.50	19.53	-13.41	94.11
stp	Standard Pressure (Millibar)	NCEI	55369	514.51	388.14	0.80	999.15
wdsp	Wind Speed (Knot)	NCEI	55369	5.08	1.73	0.90	18.14
prcp	Precipitation (Inch)	NCEI	55369	3.02	9.33	0.00	86.20
flowin	Electricity Flow-in (10^8 kWh)	NBS	55369	681.91	644.44	11.00	2334.77
flowout	Electricity Flow-out (10^8 kWh)	NBS	55369	501.15	570.52	360.00	2472.20
stnum	Charging Station	Baidu Map	55369	29028.35	40027.05	92.00	382960
r2001	Road length in 2001 (10^3 km)	NBS	55369	4.72	2.59	0.36	15.68
fossilele	Fossil fuel electricity (10^8 kWh)	NBS	55369	243.70	134.84	2.52	522.62

Notes: EDGAR (Emissions Database for Global Atmospheric Research); NBS (National Bureau of Statistics); NCEI (National Centers for Environmental Information). This table presents the descriptive statistics of the main variables used in the analysis, including the dependent variable, independent variable, control variables, and instrumental variables.

2. Heterogeneity analysis

Table S2. Energy Mix Heterogeneity

	(1)	(2)	(3)
	Clean ratio (0.0-0.2)	Clean ratio (0.2-0.5)	Clean ratio (0.5-1.0)
VARIABLES	lnco2	lnco2	lnco2
EV sales	0.0354*	0.0591	-0.00669
	(0.0189)	(0.0469)	(0.170)
Controls	Y	Y	Y
Power plants FE	Y	Y	Y
Month-by-year FE	Y	Y	Y
IV	Y	Y	Y
Observations	34,737	16,509	4,061

Notes: Robust standard errors in parentheses; Clustered at the power plants level. *** p<0.01, ** p<0.05, * p<0.10.

Table S3. Power plants’ capacity and citizens’ income heterogeneity

	(1)	(2)	(3)	（4）
	Low-capacity	High-capacity	Low-income	High-income
VARIABLES	lnCO_2	lnCO_2	lnCO_2	lnCO_2
EV sales	0.00739*	-0.00338	-0.00559	0.00959***
	(0.00443)	(0.00429)	(0.00507)	(0.00357)
Controls	Y	Y	Y	Y
Power plants FE	Y	Y	Y	Y
Month-by-year FE	Y	Y	Y	Y
Observations	21,664	33,705	27,662	27,707

Notes: Robust standard errors in parentheses; Clustered at the power plants level. *** p<0.01, ** p<0.05, * p<0.1.This table shows that small-scale plants often rely on older, less efficient technologies with higher emissions per unit of electricity generated. When EV-driven electricity demand rises, these plants may operate more frequently or at suboptimal loads, increasing their marginal emissions. In contrast, large-scale plants typically employ advanced technologies and benefit from economies of scale, achieving lower emissions intensity. Moreover, in high-income cities, the widespread adoption of electric vehicles leads to a substantial increase in electricity demand, particularly during peak charging periods.

3. Robustness check

Table S4. Results for double machine learning

VARIABLES	(1)<br>Random Forest	(2)<br>Support Vector Machine	(3)<br>Gradient Boosting	(4)<br>Neural Net
EV sales	0.0112***	0.0318*	0.0452**	0.00800***
	(0.00185)	(0.0176)	(0.0218)	(0.00171)
Controls	Y	Y	Y	Y
Power plant FE	Y	Y	Y	Y
Month-by-year FE	Y	Y	Y	Y
Observations	55369	55369	55369	55369

Notes: Robust standard errors in parentheses; Clustered at the power plants level. *** p<0.01, ** p<0.05, * p<0.10.

Table S5. Alternative emission measures

VARIABLES	(1)<br>lnN_2O	(2)<br>lnCH_4	(3)<br>lnGHG	(4)<br>lnCO_2(MEIC)
EV sales	0.0272**	0.0148	0.0232*	0.00453***
	(0.0125)	(0.0138)	(0.0119)	(0.00128)
Controls	Y	Y	Y	Y
Power plants FE	Y	Y	Y	Y
Month-by-year FE	Y	Y	Y	Y
IV	Y	Y	Y	Y
Observations	53,607	53,607	55,369	45,457

Notes: Robust standard errors in parentheses; Clustered at the power plants level. *** p<0.01, ** p<0.05, * p<0.1. In models (1) and (2), the dependent variables are N2O and CH4, both of which are sourced from the EDGAR (Emissions Database for Global Atmospheric Research). These gases are important contributors to global warming. In models (3) and (4), lnghg represents the logarithm of total greenhouse gas emissions, and lnCO2(MEIC) refers to carbon dioxide emissions data from the Multi-resolution Emission Inventory for China, which has been calibrated to more accurately reflect China's energy consumption patterns and emissions.

4. The LM tests in spatial economics

Table S6. LM test results

	Inverse distance		Binary contiguity	
Test	Statistics	P-value	Statistics	P-value
Spatial error				
Moran’s I	65.350	0.000	16.661	0.000
Lagrange multiplier	4672.160	0.000	292.996	0.000
Robust Lagrange multiplier	4472.728	0.000	291.639	0.000
Spatial lag				
Lagrange multiplier	199.455	0.000	1.437	0.231
Robust Lagrange multiplier	0.023	0.880	0.080	0.778

Notes: Moran’s I are positive and significant at 1%, indicating the spatial dependency of carbon emissions in power plants. For the spatial error model, the P-values of LM and robust LM statistics are significant ($p<1\%$). The P-values of robust LM statistics in the spatial lag model are larger than 10%. Therefore, the spatial error model is appropriate for this research. Given that the LM test for the inverse distance matrix is 199.455 ($p<1\%$), this paper lists the SAR results to examine the spatial spillover effects. According to LM tests, the Spatial Durbin Model (SDM) and the Spatial Autocorrelation (SAC) model are inappropriate because not all of the robustness LM statistics are significant.

5. Sensitivity check

Table S7. Sensitivity analysis

VARIABLES	(1) Winsorization (1%-99%) $lnCO_2$	(2) Winsorization (5%-95%) $lnCO_2$	(3) Fixed effects adjustment $lnCO_2$
EV sales	0.041*	0.039*	0.038***
	(0.021)	(0.023)	(0.009)
Controls	Y	Y	Y
Power plants FE	Y	Y	Y
Month-by-year FE	Y	Y	Y
Province-year FE	N	N	Y
IV	Y	Y	Y
Observations	55,369	55,369	55,369

Notes: Robust standard errors in parentheses; Clustered at the power plants level.*** p<0.01, ** p<0.05, * p<0.1. In Columns (1) and (2), the dependent and key explanatory variables are winsorized at the 1%–99% and 5%–95% levels, respectively. This approach reduces the potential bias arising from extreme values that may disproportionately affect the estimated coefficients, which is particularly relevant given the heterogeneity in plant-level emissions data. Column (3) introduces province-by-year fixed effects to control for time-varying unobserved heterogeneity at the provincial level.

6. Exclusion restriction of IV

Table S8. Exclusion restriction of IV

	(1)	(2)
VARIABLES	$lnCO_2$	$lnCO_2$
Charging × road	0.009***	0.008**
	(0.003)	(0.023)
EV sales		0.009***
		(0.002)
Controls	Y	Y
Power plants FE	Y	Y
Year FE	Y	Y
Observations	55,369	55,369

Notes: Robust standard errors in parentheses; Clustered at the power plants level. *** p<0.01, ** p<0.05, * p<0.1. This table estimates a reduced-form specification by regressing carbon emissions directly on the instrument (Charging × road). The coefficient on the instrument becomes smaller in magnitude and less precisely estimated once EV adoption is controlled for, while EV sales remain positive and statistically significant. This pattern suggests that the association between the instrument and emissions is largely mediated by EV adoption, providing supportive evidence for the plausibility of the exclusion restriction. Nevertheless, we acknowledge that the exclusion restriction cannot be directly tested, and thus interpret the IV estimates with appropriate caution.

7. Alternative IV

Table S9. Alternative IV

VARIABLES	First-stage<br>EV sales	Second-stage<br>$lnCO_2$
L. EV_sales × road	0.049***	
	(0.003)	
EV sales		0.030**
		(0.013)
Controls	Y	Y
Power plants FE	Y	Y
Month by Year FE	Y	Y
K-P rk LM	144.436	
K-P rk Wald F	367.617	
Observations	53,786	53,786

Notes: Robust standard errors in parentheses; Clustered at the power plants level. *** p<0.01, ** p<0.05, * p<0.1. This table constructs an alternative instrumental variable by interacting lagged EV sales with road length. This specification exploits the persistence in EV adoption and the pre-existing transport infrastructure to generate exogenous variation in current EV sales, while mitigating concerns that contemporaneous EV adoption may be correlated with unobserved factors affecting emissions. In the second stage, the estimated coefficient on EV sales remains positive and statistically significant, consistent with the baseline results.
